# A Novel Experiment-Free Site-Specific TDoA Localization Performance-Evaluation Approach

**DOI:** 10.3390/s20041035

**Published:** 2020-02-14

**Authors:** Xiaokang Ye, José Rodríguez-Piñeiro, Yuan Liu, Xuefeng Yin, Antonio Pérez Yuste

**Affiliations:** 1 College of Electronics and Information Engineering, Tongji University, Shanghai 201804, China; yexiaokang@tongji.edu.cn (X.Y.); liuyuan@tongji.edu.cn (Y.L.); 2 National Computer and Information Technology Practical Education Demonstration Center, Tongji University, Shanghai 201804, China; 3 School of Telecommunication Systems Engineering, Technical University of Madrid, Madrid 28040, Spain; antonio.perez@upm.es

**Keywords:** source localization, time difference of arrival (TDoA), performance evaluation, propagation graph, channel impulse response (CIR), universal software-defined radio peripheral (USRP)

## Abstract

Time difference of arrival (TDoA) technology is widely utilized for source localization, which stimulates many studies on performance-evaluation approaches for TDoA localization systems. Some approaches using simulations are designed merely for a simple Line-of-Sight (LoS) scenario while some other ones using experiments show high cost and inefficiency. This paper proposes an integrated approach to evaluate a TDoA localization system in an area with a complicated environment. Radio propagation graph is applied through a simulation to obtain channel impulse responses (CIRs) between a source to be located and the TDoA sensors for the area. Realistic signals received by the sensors in baseband are emulated combining the source transmitted signal and the CIRs. A hardware unit takes charge of sending the radio emulated received signals to the system under test, which is consistent with real experimental measurements. Statistical analysis of the system is allowed based on localization errors obtained comparing the system’s estimates with the ground truth of the source location. Verified results for LoS and non-LoS scenarios with variable transmitted signal bandwidths and signal-to-noise ratios, as well as for three variations of the sensor locations in an automobile circuit, show the usability of the proposed experiment-free performance-evaluation approach.

## 1. Introduction

Recently, source localization plays an increasingly significant role in detecting and tracking unlicensed devices, utilizing a series of technologies, e.g., time of arrival (ToA) [1], time difference of arrival (TDoA) [2], frequency difference of arrival, direction of arrival [3] or hybrid ones [4,5]. Among these, the TDoA technology is widely applied in consideration of a good tradeoff between performance and consumed resources for localization.

The TDoA localization for a possible radio source is generally performed with an integrated system whose sensors synchronously receive signals from the source and in-built algorithm processes the received data. Three attributes mainly influence the localization performance of a TDoA system: (a) radio wave propagation from sources to the sensors, (b) system errors or hardware limits of the sensors, and (c) localization algorithms. Firstly, a complicated environment for signal propagation is a challenge for locating a source, e.g., dense buildings in an area featuring an urban canyon environment increase signal blockages between the source and the sensors, or rich objects in an indoor area result in a multi-path phenomenon. The localization performance will excel when the signals transmitted from the source propagate to the sensors through unblocked waves. Secondly, imperfections existing in the sensors of a practical localization system can cause severe degradation of the performance, e.g., the time synchronization error among the sensors, the phase noise in the system clock. Furthermore, the hardware limits of the sensors have a constraint on the localization performance that the system can reach, e.g., the sensitivity and the maximum sampling rate of the sensors. Thirdly, the localization performance to a great extent depends on the adopted algorithm which estimates source locations processing the TDoA information obtained with the wave propagation influences, the system errors and other necessary information [6,7,8,9,10]. In summary, evaluations of the TDoA localization performance is of significance for improving localization accuracy and applicability of practical TDoA-sensing systems.

A large number of contributions have been made on the performance evaluation of TDoA localization systems [11,12,13,14,15,16,17,18,19,20,21,22,23,24,25,26,27,28,29]. They can be categorized into the simulation, the experiment and the combinations of these two.

Many researchers evaluated the performance of localization algorithms through numerical simulations in consideration of the system errors modeled as analytical expressions [11,12,13,14,15,16]. Although these studies provide closed-form solutions to improve the algorithms, they typically assume a simple scenario where only Line-of-Sight (LoS) paths may exist. While, experimental measurements in a realistic area are commonly carried out to evaluate the performance of localization systems [17,18,19,20,21,22,23]. Despite the ability to consider real wave propagation and practical system errors, the experiments show an operation inconvenience as well as high cost, and the measurement results obtained in one area can not be applied to another. Other approaches integrating the simulation and experiment [24,25,26,27,28,29] wisely apply statistical radio channel models obtained from real channel measurements to the simulation-based performance evaluation, or provide the statistical characteristics of algorithms or systems through Monte Carlo simulations before conducting experiments. However, the hybrid approaches display the inapplicability of providing a practical localization system with deterministic information of the wave propagation for a specific area. In Section 2, some of the aforementioned contributions will be revisited, in order to show the details of their pros and cons. The differences between our approach and those studies will be also highlighted.

We propose an enlightened thought to the performance evaluation for TDoA localization systems. In our approach, the radio propagation graph is utilized to obtain the deterministic wave propagation information in reality for an area of interest. Moreover, a hardware Radio-Frequency (RF) unit serves as transmitting radio signals to the system under test. In this way, the proposed approach can be understood as a localization performance-evaluation platform based on a hardware-in-loop idea that makes use of the propagation graph to emulate the signals that the TDoA sensors are expected to receive in the area.

The main novelties and contributions of our work lie in:

1. For an area with a complicated environment, deterministic information of the wave propagation between the source to be located and the sensors is able to be extracted using the radio propagation graph. The wave propagation information offers an emulator of signals propagating in the area consistent with experimental measurements;
2. The performance evaluation for a practical TDoA localization system can be made in a laboratory without making experimental trials in a realistic area. The performances of different localization systems can be fairly compared in a cost-efficient way and in a “blind” mode, which implies that a system under test is considered as a black box without prior knowledge of any detail regarding its workflow;
3. Based on the results of wave propagation information for an area, statistical performance analysis of the system under test can be made in terms of localization errors. A root mean square and a Cumulative Distribution Function (CDF) of localization errors are obtained for a source to be located. Moreover, a geographical distribution of localization errors versus source locations for the area is presented. Especially, this kind of geographical distribution is beneficial to the deployment of multiple sensors for the system under test in the area;
4. In order to transmit radio signals synchronously to the localization system under test, the multi-link hardware-in-loop structure with accurate time synchronization among devices is utilized for the first time in the performance evaluation.

The remainder of the paper is structured as follows. The reviews on the performance-evaluation approaches for TDoA localization algorithms and systems are elaborated in Section 2. In Section 3, the proposed performance-evaluation approach is described in detail. In Section 4, the evaluation of multi-device time synchronization used in the approach is introduced. In Section 5, the propagation graph in the approach is verified through numerical simulations using two TDoA algorithms. In Section 6, the proposed approach is verified using a commercial TDoA localization system, and also the localization performance of the system is illustrated with regard to the sensor deployment for a realistic area. Finally, conclusive remarks are addressed in Section 7.

## 2. Related Work

In this section, we will elaborate on some studies in terms of their advantages and disadvantages, and compare them with our proposed work.

### 2.1. Using Simulations

In [11], the performances of six TDoA localization algorithms were evaluated through simulations under an LoS propagation condition in a 3D space. The study compared all the algorithms in terms of an average localization error, a so-called failure rate as well as a distribution map of localization errors versus source locations. In [12], two recursive TDoA algorithms were also evaluated via simulations in consideration of a random uncertainty in the sensor locations for the case of the LoS propagation between the sources and sensors. The localization performance was evaluated using an average range error and a standard deviation of localization errors. Compared to the work in [11,12], our approach not only can consider complicated propagation environments, but also can obtain the similar average and distribution map of localization errors verified from a practical TDoA system rather than a simulation.

In [13], a novel TDoA algorithm was proposed assuming that the time synchronization among sensors and the sensor locations owned random errors. The closed-form solution of the algorithm was theoretically obtained and was evaluated through system-level simulations without consideration of the wave propagation in reality. This sort of performance-evaluation approach is also found in [16]. In [14], a TDoA algorithm was investigated in the presence of the clock offset and skew in the sensors. The algorithm was theoretically derived and was evaluated through simulations without taking the wave propagation into account. Moreover, the evaluations in this study were performed with only few configurations of the sensor and source locations. In contrast to the studies in [13,14,16], our approach shows the feasibility of considering the wave propagation information for an area in reality for the performance evaluation.

In [15], a recent TDoA algorithm proposing a closed-form solution was verified via Monte Carlo simulations utilizing a mean square and a CDF of localization errors as performance-evaluation metrics. The evaluations were independent of source locations by generating a random one for each simulation run. Compared to this, we construct a distribution of localization errors versus source locations providing discussions on the sensor deployment of the system for an area.

### 2.2. Considering Experimental Validation

In [17,18], two commercial TDoA localization systems were proposed and mounted in the area around an airport for surveillance purposes. Their performances were evaluated with experimental measurements in two scenarios where fixed and moving targets were located in the area respectively. In [19], an Ultra-Wide-Band (UWB) TDoA localization system utilized for an automobile parking system was exhibited. A mean square localization error was used as a metric to evaluate the localization system for a moving source. The above three studies introduced the performance evaluation of locating moving sources using experiments. Although the performance evaluation for a moving source is not currently implemented in our paper, it is not difficult to realize it using the same framework of the proposed approach.

In [20], another UWB system with a time-synchronization technique of reference tag was introduced for the localization in an indoor area. Only LoS experimental measurements were conducted in this study to evaluate the system performance, and the CDFs of localization errors were obtained from the measurements. In [22], an UWB TDoA/ToA localization system utilizing a technique of wireless time synchronization was evaluated for the performance in an indoor area under an LoS condition. A metric of so-called circular error probability was utilized for the performance evaluation. In [23], a recent TDoA localization technology without the time synchronization among the sensors was implemented in an indoor localization system. The technology was evaluated through experiments in a factory-like environment under an LoS condition, and the CDFs of localization errors were extracted from the measurements. The investigations in [20,22,23] performed experimental measurements only in LoS environments rather than more complicated ones. Instead, our approach has the feasible ability to provide the performance evaluation for LoS, Non-LoS (NLoS) and other multi-path scenarios.

### 2.3. Using Combinations of Simulations and Experimental Validation

In [24], in order to generate noise profiles of TDoA measurements in the simulations, a statistical channel model characterizing delay spread based on real channel measurements was selected for a suburban environment. In [28], an impact of the numbers of clusters and rays in an environment was investigated on the localization accuracy for LoS and NLoS scenarios. Such a way can be useful in the aspect that a typical environment, in reality, can be taken into account. Although these two studies skillfully integrated the influences of signal delays on TDoA measurements into the performance evaluation, it can not provide deterministic delay information for an area of interest if one would like to know the exact propagation delay for each wave path component.

In [25], radio channel simulations were performed to obtain power delay profiles and radio channel parameters which were utilized as the input of the algorithms to be evaluated. This evaluation flow is quite similar to ours. However, our proposed approach does not utilize the statistical models in the radio channel simulations. Instead, the propagation graph in our case can directly generate the channel impulse responses (CIRs) in a deterministic way, which more accurately describes how the wave propagates in the area. In [27], a path loss model featuring an urban-macro-cell-LoS environment was exploited in the radio channel simulation for the algorithm evaluation. However, the fading model was unable to provide the propagation delay of the signals utilized by the TDoA technique.

In [26], a TDoA algorithm alleviating errors caused by NLoS wave propagation was evaluated through both simulation and experiment. The evaluations were performed setting some of the links between a source and the sensors as NLoS ones which were simply regarded as total blockages. Such an operation seems a good trial of considering the influence of wave propagation on the localization performance. However, this study did not further elaborate on how to describe other kinds of multiple path components, e.g. reflected paths, besides the obstructed LoS paths in an area with a complicated environment.

To the authors’ best knowledge, there exist few papers concentrating on the approaches of evaluating TDoA localization systems with obtaining wave propagation information for a specific area with a complicated environment while without carrying out experiments in the area.

## 3. Approach Architecture

### 3.1. General Structure

This paper focuses on the TDoA localization performance evaluation for stationary sources transmitting radio signals. The proposed approach consists of three components. One component is a software processing unit including the propagation graph simulation for an area as well as the baseband signal construction obtaining emulated received signals of the TDoA sensors. Another component is a hardware transmitting unit implemented using a set of RF devices, sending radio signals to a TDoA localization system under test. The other component is a performance analyzing unit including the localization error calculation and the statistical performance evaluation.

**Figure 1:**
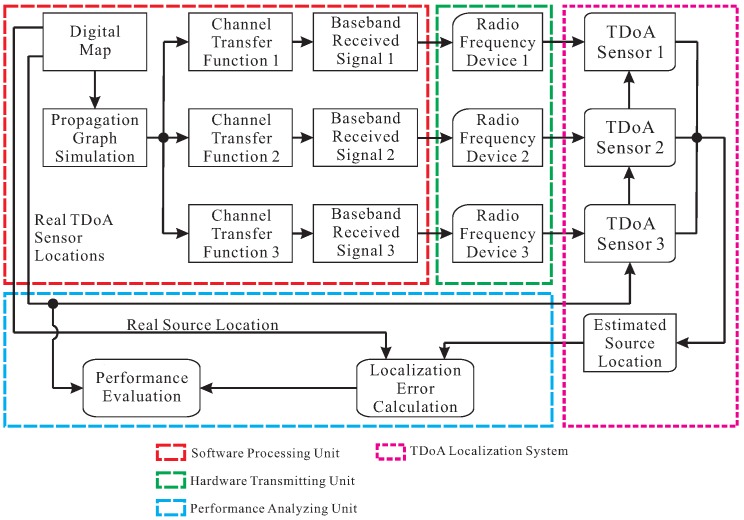
The block diagram of the proposed performance-evaluation approach for a time difference of arrival (TDoA) localization system.

Figure 1 introduces the framework of the proposed performance-evaluation approach for a commercially available TDoA localization system.

In the framework, three TDoA sensors are assumed for the localization system. One can extend the number of RF devices in line with a TDoA localization system with more than three sensors without changing the approach structure. Meanwhile, the numbers of Channel Transfer Functions (CTFs) and baseband received signals are supposed to be correspondingly extended.

**Figure 2:**
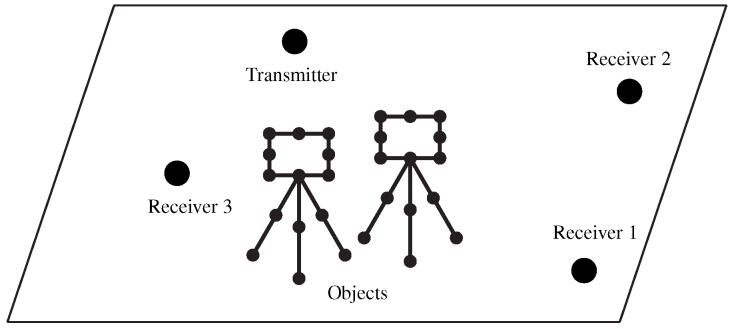
An example of digital map used in the proposed performance-evaluation approach.

Figure 2 displays an example of a digital map for an area where there exists one transmitter, three receivers and two objects.

The digital map was constructed for an area of interest in a rectangular coordinate system where the objects are discretized into scatterer vertices. Supposing a vertex is referred to as the origin of the coordinate system, the coordinates of all the other vertices can be obtained accordingly. A source to be located and the three TDoA sensors were regarded as the transmitter and three receivers, respectively, and also viewed as vertices in the digital map. The location coordinate of the transmitter is used as the ground truth of the source location. The location coordinates of the receivers are input as the sensor locations (Localization systems commonly utilize a geographic coordinate system including latitude, longitude and height. In such a case, an equivalent coordinate transformation will be performed.) to the localization system under test, and also utilized for the statistical performance evaluation. The propagation graph simulation is performed based on the constructed digital map to obtain CTF/CIR between the transmitter and each receiver.

TDoA sensors detect radio signals in the process of locating a source. The aim of the proposed performance-evaluation approach is to construct the radio “received signals” that emulate the signals detected by the sensors. In Figure 1, the three links of the received signals in baseband are obtained by multiplying the transmitted signal of the source respectively with the three CTFs from the propagation graph simulation in the frequency domain. Then, the received signals in baseband are converted into the radio signals using the RF devices. Eventually, the radio signals are transmitted to the sensors through identical cables (Here, the term “identical” has two meanings: first, the lengths of the three cables are equal. Second, the transfer functions of the cables are calibrated from the transmitted radio signals.), in order to affect the received signals per sensor as less as possible. In such a way, the sensors may virtually receive a radio version of the signal in baseband integrating the transmitted signal of the source and the CTFs.

In Figure 1, the TDoA localization system outputs the estimates of the source location after receiving the radio signals based on the same receiver locations as in the digital map. So far, the proposed hardware-in-loop performance-evaluation approach makes a realistic process of the source transmitting signals, the sensors receiving signals and the localization system estimating the source location, which is consistent with the experimental measurements. The localization errors are calculated by comparing the ground truth and the estimates of the source location. More importantly, our proposed approach provides statistical performance analysis based on the localization errors for the system improvements.

### 3.2. The Software Processing Unit

#### 3.2.1. Generation of Multi-Link Propagation Channels

The propagation graph theory was initiated in [30] to obtain CIRs in a stochastic way, which is unable to attain the specific coefficients for different wave propagation mechanisms. However, a later study in [31] issued an approach of obtaining CIRs by combining a Ray-Tracing (RT) technique and a modified deterministic propagation graph. The former is to obtain specular components (Here, the specular components are referred to as the ones caused by the wave reflection and diffraction.) in the propagation channel while the latter is to obtain Diffuse Scattering (DS) ones. The propagation graph used in our proposed performance-evaluation approach is modified based on [31], in which the specular components are obtained using the propagation graph instead of the RT since the propagation graph takes less computational complexity.

In the aforementioned digital map, an edge between two vertices is regarded as “visible” if the wave can propagate directly from one vertex to another. A feasible propagation path may be composed of multiple edges for the wave reflection and diffraction. Assuming that a TDoA localization system with $M_{r}$ sensors is utilized to locate $M_{t}$ sources in an area where $M_{s}$ scatterer vertices are placed, the complete CTF denoted by H(f) can be calculated as

(1) $$H\left(f\right)=D\left(f\right)+\sum_{j=1}^{J}F_{j}\left(f\right)+\sum_{k=1}^{K}S_{k}\left(f\right)+R\left(f\right){\left[I-B(f)\right]}^{-1}T\left(f\right),$$

where $I\in C^{M_{s}\times M_{s}}$ and ${\left(\cdot \right)}^{-1}$ denote the identity matrix and the inverse of a given matrix, respectively. The matrices $D\left(f\right)\in C^{M_{r}\times M_{t}}$, $F_{j}\left(f\right)\in C^{M_{r}\times M_{t}}$, $S_{k}\left(f\right)\in C^{M_{r}\times M_{t}}$, $T\left(f\right)\in C^{M_{s}\times M_{t}}$, $B\left(f\right)\in C^{M_{s}\times M_{s}}$ and $R\left(f\right)\in C^{M_{r}\times M_{s}}$ denote the transmission matrices, where an entry represents the CTF of a propagation path denoted by ε for the LoS component, the *j*-bounce reflection and the *k*-bounce diffraction from transmitter to receiver, as well as the DS from transmitter to scatterer, from scatterer to scatterer and from scatterer to receiver. *J* and *K* represent the maximum numbers of reflection and diffraction bounces, respectively, and *f* denotes the frequency.

The CTF of the propagation path ε can be calculated as

(2) $$H_{\varepsilon }\left(f\right)=g_{\varepsilon }\left(f\right)\exp\left(-j2\pi \tau_{\varepsilon }f\right),$$

where $g_{\varepsilon }\left(f\right)$ and $\tau_{\varepsilon }$ denote the propagation gain and the propagation delay for the path ε respectively. The propagation gains for D(f), T(f), B(f) and R(f) are explicitly stated in [31]. In this paper, the propagation gains for $F_{j}\left(f\right)$ and $S_{k}\left(f\right)$ are elaborated as $\frac{c}{4\pi r_{m}f}\cdot P^{j}$ and $\frac{c}{4\pi r_{n}f}\cdot Q^{k}$, respectively, *c* denotes the light speed. $r_{m}$ and $r_{n}$ denote the total propagation distances respectively along the reflection and the diffraction path from transmitter to receiver. *Q* is the diffraction loss coefficient. The reflection loss coefficient *P* is calculated based on the scattering loss coefficient *S* as [32]

(3) $$P=\sqrt{1-S^{2}},$$

where *S* ranges from 0 to 1. In our case, the CTFs for the area are obtained from LoS, reflection, diffraction and DS path components. Their contributions are calculated separately, i.e., only one sort of propagation mechanism is considered for a path component. The composite CTF for the area is obtained superposing the four parts of the path components.

#### 3.2.2. Generation of Multi-Link Received Signals

An arbitrary digital sequence is generated [33], and then it passes through a Raised-Cosine (RC) filter [34], i.e., the responses of the digital sequence and the RC filter are multiplied in the frequency domain. The noise-free received signal is constructed combining the RC-filtered signal and the CTFs obtained from the propagation graph simulation [35], denoted by $\tilde{y}\left(f\right)$. In order to imitate the received signals contaminated by noise, zero-mean complex circularly-symmetric white Gaussian noise is added to the signal $\tilde{y}\left(f\right)$ with a specific Signal-to-Noise Ratio (SNR) [36]. The SNR denoted by $r_{e}$ is calculated as

(4) $$r_{e}=\frac{\frac{1}{W}\int_{-W/2}^{W/2}{\left|\tilde{y}\left(f\right)\right|}^{2}df}{P_{n}},$$

where *W* and $P_{n}$ denote the frequency bandwidth (BW) and the noise power, respectively.

### 3.3. Hardware Transmitting Unit

#### 3.3.1. Construction Structure

**Figure 3:**
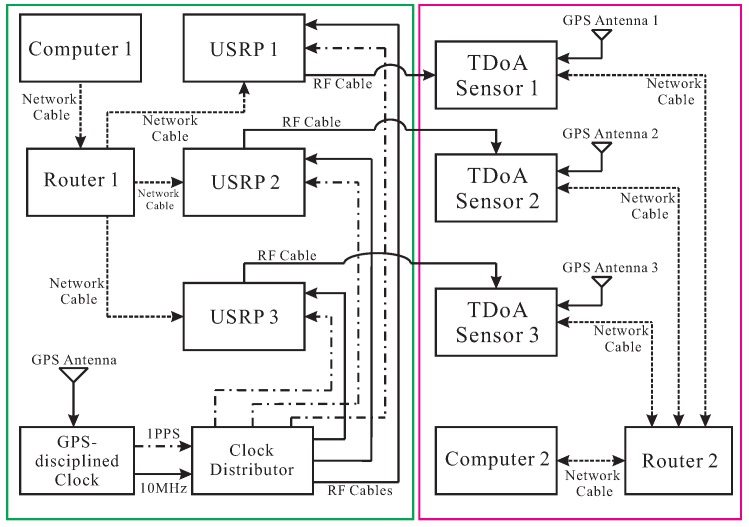
The block diagram of the hardware part of the proposed performance-evaluation approach for a TDoA localization system. The left (green box) and the right (magenta box) sides show the hardware part of the approach and an example of TDoA localization system under test, respectively.

Figure 3 illustrates the hardware part of the approach implemented based on the framework in Figure 1.

In Figure 3, the hardware transmitting unit is composed of the three off-the-shelf universal software-defined radio peripherals (USRPs) [37] transmitting radio signals (“USRP 1”, “USRP 2” and “USRP 3”), a host computer controlling the USRPs (“Computer 1”), a router connecting the host computer and the USRPs (“Router 1”), as well as a hardware suite for the time synchronization, i.e., “Global Positioning System (GPS) Antenna”, “GPS-disciplined Clock” and “Clock Distributor”. The propagation graph simulation and the emulated received signal generation are also performed in the Computer 1. The router is used to construct a wired Local Area Network (LAN) where the Computer 1 can transmit commands and data through the network links. The three USRPs convert the signals in the baseband to radio signals on a carrier with a specific frequency. The carrier frequency set in the TDoA localization system under test is configured the same as all the USRPs. Each sensor of the localization system is connected to a USRP with a cable via an RF port. The lengths of the three cables are identical to avoid as much as possible the differences in the delay of the signals received by the system. Moreover, the transfer functions of these cables are known.

Moreover, an example of a TDoA localization system to be tested is also illustrated in Figure 3. A host computer (“Computer 2”) and the three TDoA sensors constitute a wired LAN using a router (“Router 2”), so that one can control the three sensors by the Computer 2. Figure 3 also shows the time synchronization among the three sensors of the system in virtue of exposing a GPS antenna for each sensor to the sky (“GPS Antenna 1”, “GPS Antenna 2” and “GPS Antenna 3”). Note that the time synchronization approach used by the localization system is unknown to us.

#### 3.3.2. Time-Stamp-Based Technique and Synchronization Implementation

The time synchronization among all the sensors of the TDoA localization system for receiving radio signals is of great importance to the localization performance [38]. It demands that the hardware transmitting unit in the proposed performance-evaluation approach should be configured with accurate time synchronization, which implies that all the USRPs transmit the radio signals at the same time moment.

As shown in Figure 3, the GPS-disciplined clock provides “one pulse per second” (1PPS) square-wave and 10-MHz sinusoidal-wave signals repeatedly utilizing the received GPS signals from the GPS antenna. The clock distributor [39] outputs duplicated 1PPS and 10-MHz square-wave signals, respectively. The 1PPS signal establishes a common time reference to send commands to the USRPs as well as to start and stop the transmission of the signals simultaneously. The 10-MHz signal provides a common external reference for the oscillators of all the USRPs, in order to obtain an identical phase among them as far as possible [38].

**Figure 4:**
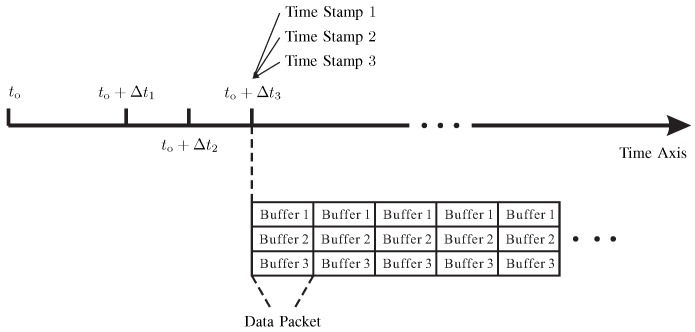
The time axis for configuring the universal software-defined radio peripherals (USRPs) parameters and transmitting the data packet for the time synchronization method in the proposed performance-evaluation approach.

In our approach, the time synchronization among all the USRPs is implemented using a custom-developed software with the Boost [40] and the Ettus USRP hardware driver [41]. Figure 4 depicts the USRP parameter configurations and the data transmission along the time axis.

The key steps of the time synchronization are described in the case of three USRPs as follows.

1. The 1PPS signal is utilized to establish a common time basis for all the three USRPs. Synchronously with a 1PPS pulse, the origin of the time basis for all the USRPs is established, i.e., all the USRPs are synchronized with a 1PPS pulse at the time $t_{o}$. This common time basis will be used in all subsequent steps to perform operations in a synchronous way among different USRPs.
2. Set the carrier frequency and the transmitting sampling rate at the time instant $t_{o}+\Delta t_{1}$ and $t_{o}+\Delta t_{2}$ for all the USRPs, respectively.
3. A memory buffer is created for each USRP in the memory of the computer to store the data samples to be sent by each USRP during a specific time period.
4. The data samples are temporarily copied into the memory buffers. A time stamp is built for the data in each buffer, i.e., “Time Stamp 1”, “Time Stamp 2” and “Time Stamp 3” in Figure 4, in order to determine the exact time instant at which the transmission starts. Then a data packet is constructed including all the buffers.
5. The first data packet starts to be transmitted at the time instant $t_{o}+\Delta t_{3}$. In other words, the three time stamps are set identical and equal to $t_{o}+\Delta t_{3}$ for the first data packet. The radio signal is transmitted continuously by setting the sending time of the subsequent data packets properly, i.e., there is no time spacing between two adjacent data packets. Note that $\Delta t_{1}$, $\Delta t_{2}$ and $\Delta t_{3}$ are not necessarily integer times of one second. $\Delta t_{1}$, $\Delta t_{2}$ and $\Delta t_{3}$ are set as 4, 2 and 14 seconds, respectively, in practice for our case.

In summary, a common time source provided by the aforementioned GPS-disciplined clock is shared by all the RF devices in the proposed time synchronization method. By using this way, all the RF devices can be aligned with each other for the same time moment using the “time-stamp” technique.

### 3.4. Performance Analyzing Unit

#### 3.4.1. Localization Error Calculation

Localization error is a common metric for measuring the localization performance. The localization error denoted by ρ can be calculated as

(5) $$\rho ={∥\hat{r}-r∥}_{2},$$

where $\hat{r}=\left(\hat{x},\hat{y},\hat{z}\right)$ and r=(x,y,z) denote an estimate by the localization system and the ground truth of the source location in the 3D coordinate system, respectively. ${∥\cdot ∥}_{2}$ represents the 2-norm of the given argument. Similarly, Equation (5) can be also used to calculate the localization error in 2D horizontal plane, where $\hat{r}=\left(\hat{x},\hat{y}\right)$ and r=(x,y).

#### 3.4.2. Statistical Performance Evaluation

To statistically measure the localization errors for a source in an area, a certain number of noise realizations are utilized to obtain a Root Mean Square Localization Error (RMSLE). The RMSLE denoted by $\bar{\rho }$ is calculated as

(6) $$\bar{\rho }={\left(\frac{1}{N_{s}}\sum_{n=1}^{N_{s}}\rho_{n}^{2}\right)}^{\frac{1}{2}},$$

where $N_{s}$ denotes the number of realizations. Moreover, a CDF of all the realizations of localization errors can be also illustrated to investigate statistical characteristics of the localization system under test, e.g., a median localization error, a localization error owned by 90% realizations.

Besides the RMSLE for one source, another statistical investigation is performed by placing a certain number of sources in the area. The RMSLE is obtained for each source candidate in the area. As a result, a “localization error map” is generated mapping the RMSLEs of all the sources to their respective geographical locations (hereafter referred to as “RMSLE heat map”).

Note that there exists a standard from International Organization for Standardization (ISO) called “ISO 18305” featuring localization performance metrics. The standard also proposes other error functions and metrics besides the localization error ρ. However, in this work, our intention is to generally give a novel idea of localization performance-evaluation approach based on simulation studies that have an advantage of ergodicity when testing over all possible source candidates. Through some manipulations, the localization error ρ can be extended to include other error functions and metrics as those suggested in the ISO 18305.

## 4. Performance Evaluation for Time Synchronization

The accuracy of the time synchronization in the proposed approach is evaluated using an oscilloscope. The procedure of evaluating the time synchronization is described as follows: (a) Connect each USRP to the oscilloscope with a cable through an RF port. The lengths of the three cables are identical to avoid as much as possible the differences in the delay of the signals received by the oscilloscope. Moreover, the transfer functions of these cables are known. (b) Set a carrier frequency for the three USRPs. Then the signal with constant magnitude valued as “1” in baseband is transmitted on the radio carrier from the USRPs using the proposed time-stamp-based synchronization technique. (c) Trigger the oscilloscope simultaneously so that the three RF channels of the oscilloscope can collect the signals at the same time. Then the waveforms can be observed and the waveform data is recorded. We denote the carrier frequency as $f_{c}$. The radio signal emitted by the USRP in the time domain, denoted by s(t), can be expressed as

(7) $$s\left(t\right)=s_{I}\cdot \cos\left(2\pi f_{c}t\right),$$

where $s_{I}=1$ represents the real part of the signal in baseband. Therefore, a sinusoidal wave with the frequency of $f_{c}$ is supposed to be observed.

**Figure 5:**
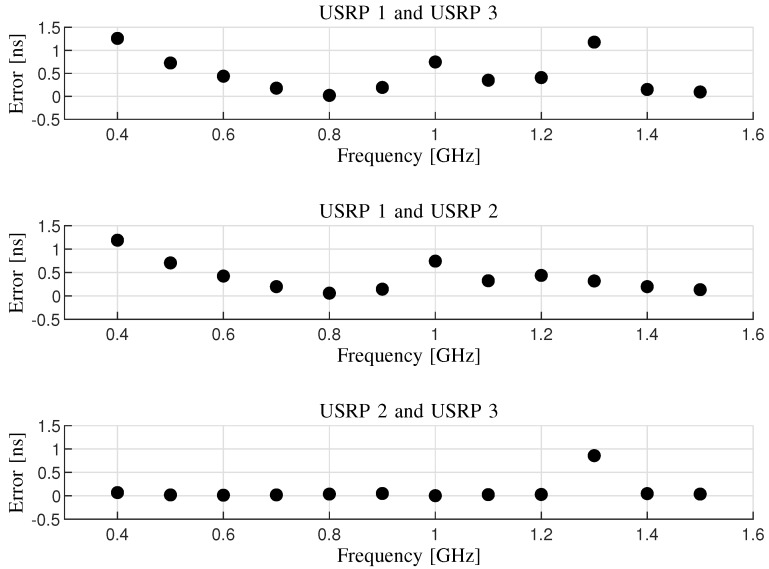
The time synchronization errors among the USRPs versus the frequency tested using the oscilloscope.

Figure 5 shows the empirical results of the synchronization errors with multiple tests.

The time differences between arbitrarily two of the three USRPs are illustrated versus a frequency range from 0.4 to 1.5 GHz with 0.1 GHz spacing. Note that the sampling rate of the oscilloscope is 10 GSa/s. Hence, the resolution for the synchronization error can be calculated as the inverse of the sampling rate, i.e., 0.1 ns.

It can be observed from Figure 5 that the maximum error of the time synchronization for the specific frequency band is less than 1.5 ns. In [42], a real-time TDoA localization system was proposed, and the time synchronization using a technique of a reference clock has a synchronization error of 10 ns. In [43], the synchronization of a practical localization system was performed utilizing a motion sensor and an external real-time clock. The maximum error is about 7.99 ns in this study. In [44], the time synchronization was implemented making each sensor of the localization system configured with a GPS antenna. The synchronization error is about 40 ns in this paper. It implies that the error is higher using separate GPS sources than a common GPS source for all the sensors of the system. Therefore, the proposed time synchronization method is reliable to providing a benchmark for the localization system under test.

## 5. Propagation Graph Verification

In this section, the propagation graph simulation of the proposed TDoA localization performance-evaluation approach is verified using two TDoA localization algorithms.

### 5.1. Propagation Graph Implementation

**Figure 6:**
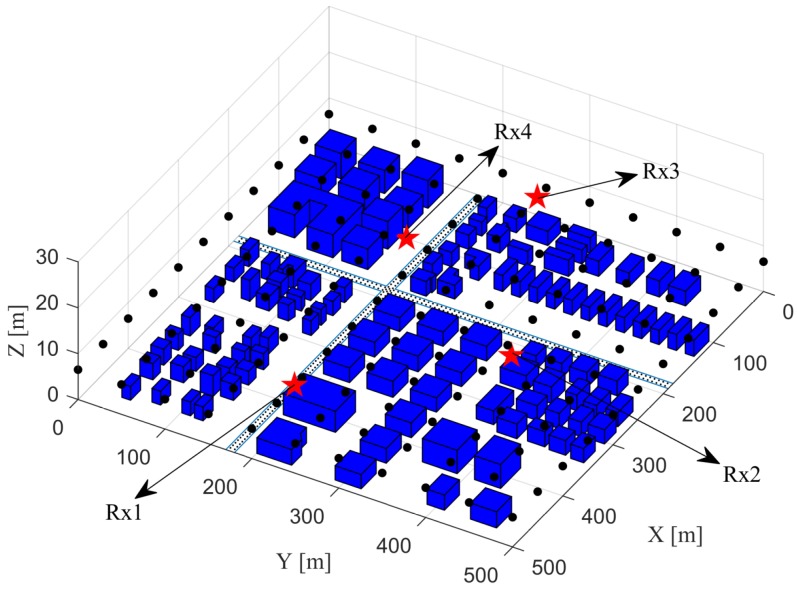
The area used for the verification of the propagation graph simulation of the proposed performance-evaluation approach. The 111 objects marked in blue are placed in this area.

Figure 6 displays a 500 m × 500 m × 30 m area to be loaded as a digital map, for the verification of the propagation graph simulation of the performance-evaluation approach.

Four TDoA sensors marked with pentagrams are regarded as receivers. 121 sources to be located marked with dots are regarded as transmitters. For the coordinate system of the area shown in Figure 6, the location coordinates of the four receivers, labelled with “Rx1”, “Rx2”, “Rx3” and “Rx4”, are (415,200,6.5) m, (260,360,6) m, (35,260,9) m, and (105,150,0.8) m, respectively. The heights of all the transmitters from the XoY plane are 6.5 m. All the transmitters are uniformly located in the area, and the spacing between two adjacent transmitters is 50 m along both X and Y axes.

One assumption should be noted for the propagation graph simulation here: only five types of propagation paths are considered, i.e., LoS path, one-bounce reflection path from a transmitter to a receiver, diffraction path from a transmitter to a receiver interacting with the top edges of the objects, DS path from a transmitter to a scatterer and from a scatterer to a receiver. The parameters configured for the propagation graph simulation here are summarized in Table 1.

According to Equation (3), the reflection loss coefficients *P* for smooth and rough surfaces are respectively equal to 0.9367 and 0.8352 based on the scattering loss coefficients shown in Table 1. The diffraction loss coefficient of *Q* is obtained based on [45]. Zero-mean complex circularly-symmetric additive white Gaussian noise is added to the CIRs obtained from the propagation graph simulation.

### 5.2. TDoA Localization Algorithm Implementation

**Figure 7:**
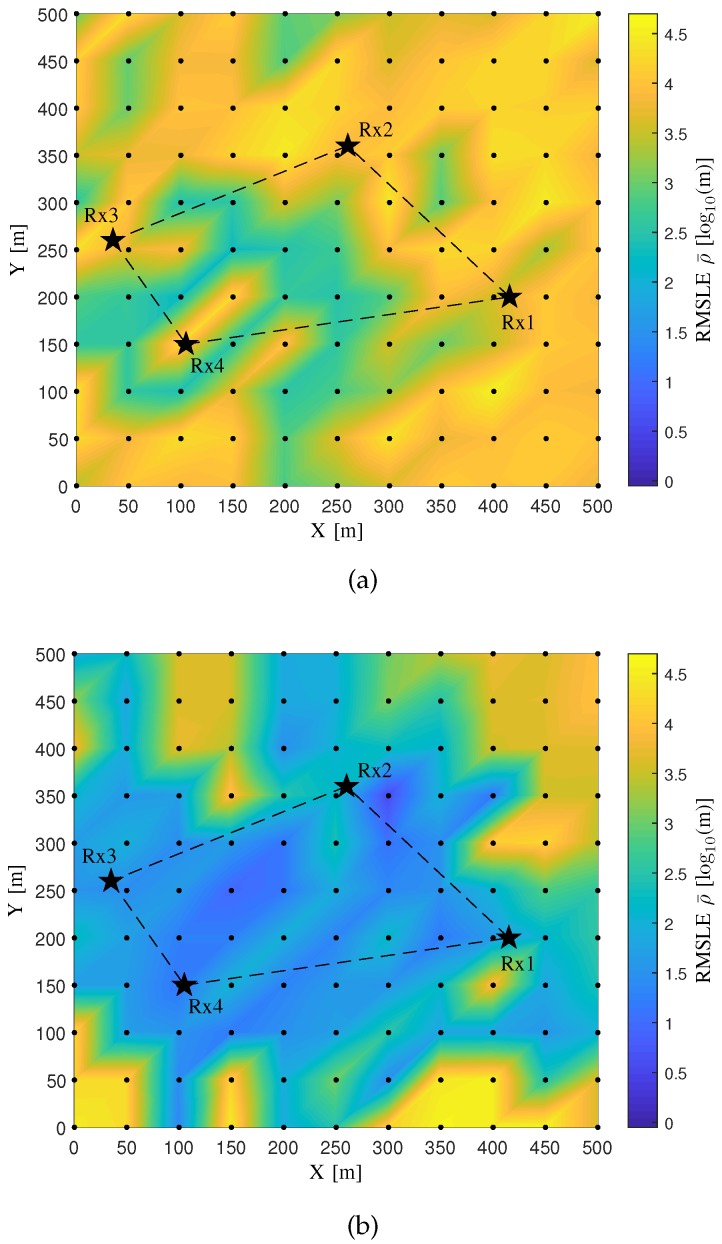
The comparison of the Root Mean Square Localization Error (RMSLE) heat map between two localization algorithms for the verification of the propagation graph simulation. (**a**) The Algorithm A; (**b**) The Algorithm B.

Two algorithms respectively using Spherical-Intersecting [46] and Levenberg–Marquart [47] techniques are implemented utilizing the CIRs obtained from the propagation graph as the input of the TDoA information. Let us denote the two algorithms as “Algorithm A” and “Algorithm B”, respectively. Specifically, in each simulated CIR for the link between a source and a sensor, the propagation delay of the path with the maximum power gain is obtained, and then the TDoA for each pair of links between the source and two sensors is attained subtracting one corresponding delay from another. For Algorithm B, the initial guess of source location for all the candidates in the area is obtained averaging each coordinate component of all the four sensor locations. Figure 7 illustrates the comparison of the RMSLE heat maps between the two algorithms.

In Figure 7, the RMSLE heat map of Algorithm B owns lower RMSLEs for the most source candidates in the area than Algorithm A. The Algorithm A is a linear solution for source localization while the Algorithm B is a non-linear one using an iterative technique. The latter algorithm outperforms the other, possibly since it has a better fitting of the observed TDoA information with the built cost-function model, and also it starts the iteration from a reasonable initial guess of the source location. Moreover, both algorithms show lower RMSLEs for the sources in the area enclosed by all the sensors than outside the area.

## 6. Performance-Evaluation Approach Verification

In this section, the proposed TDoA localization performance-evaluation approach will be verified using a commercial off-the-shelf TDoA localization system.

### 6.1. Verification for LoS and NLoS Scenarios

**Figure 8:**
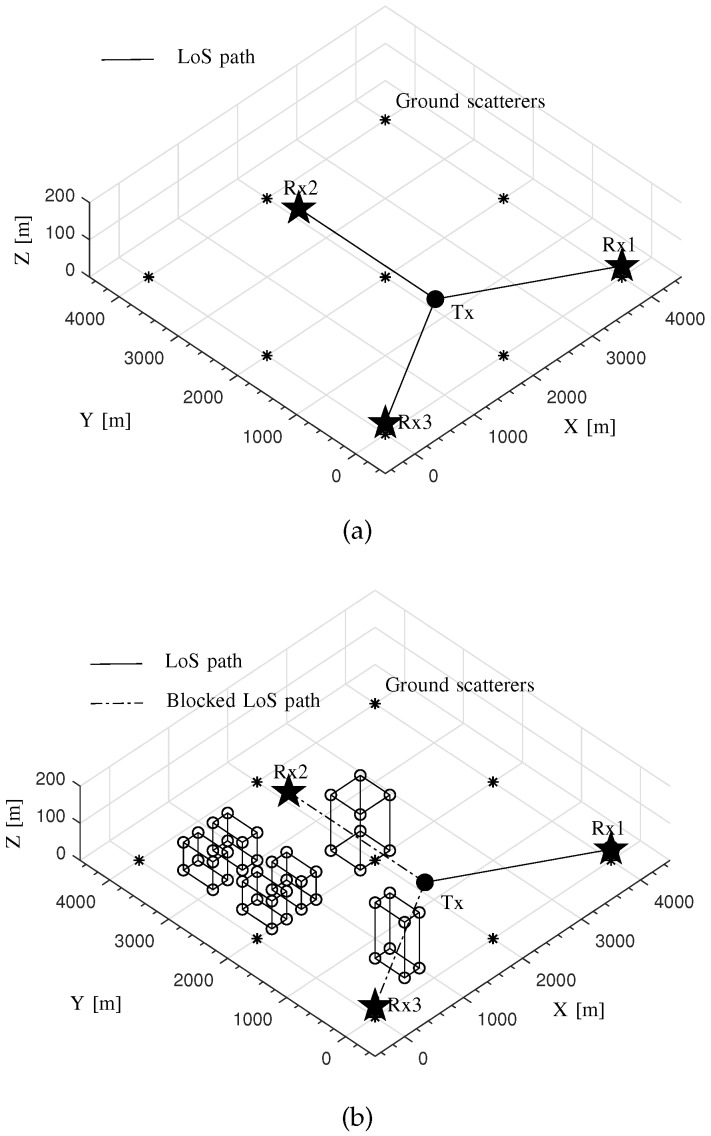
The digital maps for the Line-of-Sight (LoS) and the Non Line-of-Sight (NLoS) scenarios for the verification of the proposed performance-evaluation approach using a commercial TDoA localization system. (**a**) The LoS scenario; (**b**) The NLoS scenario.

LoS and NLoS scenarios are utilized to verify the proposed performance-evaluation approach with the transmitted signal BW and the SNR being variable. Figure 8 depicts the digital maps for these two scenarios.

In Figure 8, the source to be located and the three sensors are regarded as a transmitter and three receivers in the propagation graph simulation, labeled with “Tx”, “Rx1”, “Rx2” and “Rx3”, respectively. The 2D localization is performed for the source using the three sensors. For the coordinate system of the area, the location coordinates of Tx, Rx1, Rx2 and Rx3 are $\left(2,2/\sqrt{3},0.03\right)$ km, (4,0,0.03) km, $\left(2,2\sqrt{3},0.03\right)$ km, (0,0,0.03) km, respectively. Moreover, the scatterers on the ground (Here, the “ground” is referred to as the XoY plane in the 3D coordinate system of the area. The ground scatterers are with rough surfaces.) marked with stars in Figure 8 are also considered in the propagation graph simulation here.

For these two scenarios, we only consider LoS paths from transmitters to receivers and DS paths in the propagation graph simulation. In Figure 8a, an LoS path exists between the source and each sensor. In Figure 8b, the LoS paths between Tx and Rx2 as well as between Tx and Rx3 were blocked by the objects while the LoS path between Tx and Rx1 was still unobstructed. Moreover, some objects are placed beside Rx2 and Rx3. The signals transmitted from Tx are likely to arrive at Rx2 and Rx3 along the DS paths. For the propagation graph simulation here, the scattering loss coefficients *S* for smooth and rough surfaces were set as 0.2 and 0.6, respectively. The parameter dS was set as 0.1 and 0.4 m${}^{2}$ for smooth and rough surfaces, respectively. The localization system was tested with three BWs, i.e., 5, 25, 3125 KHz, as well as four SNRs, i.e., −3, 8, 17, 30 dB. The carrier frequency is set as 1 GHz.

**Figure 9:**
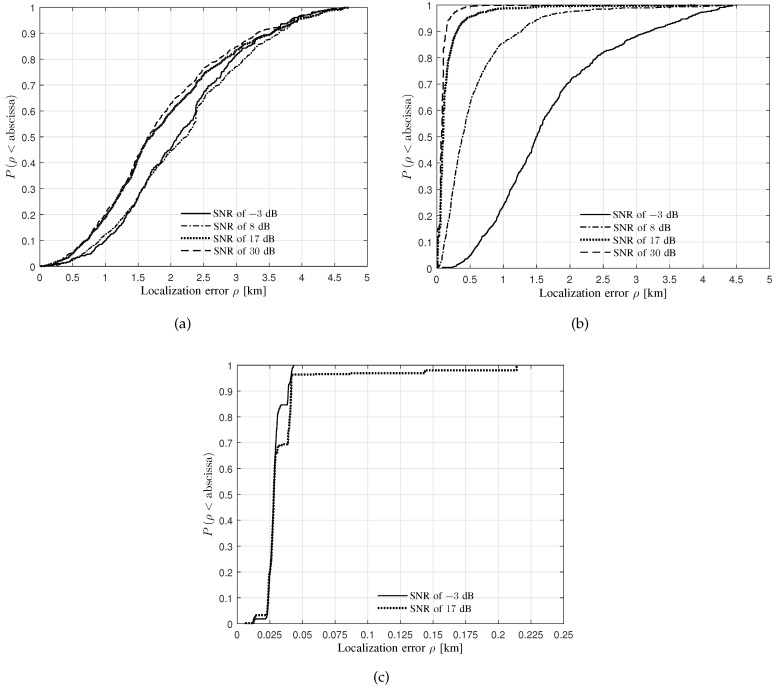
The Cumulative Distribution Functions (CDFs) of localization error for the LoS scenario obtained from the verification of the proposed performance-evaluation approach using the commercial localization system. (**a**) BW of 5 KHz; (**b**) BW of 25 KHz; (**c**) BW of 3125 KHz.

**Figure 10:**
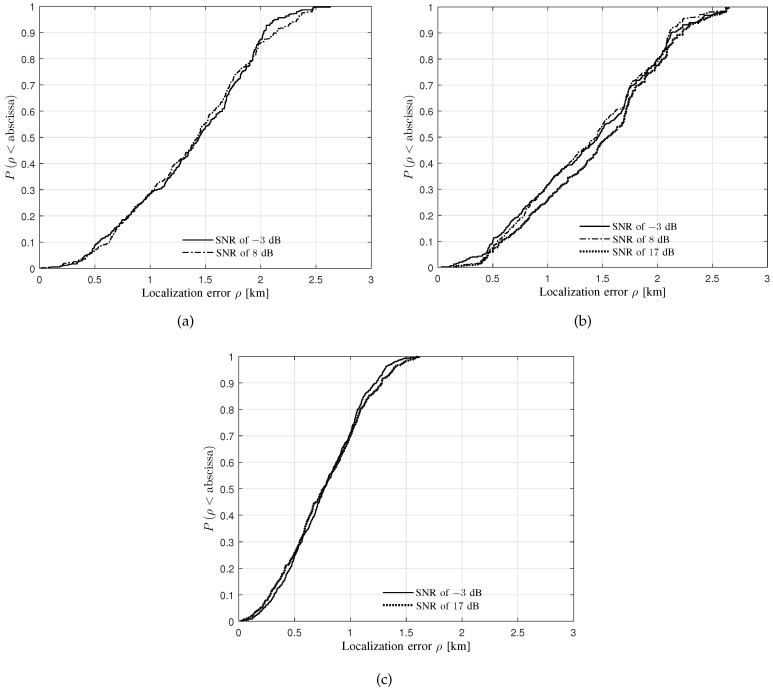
The CDFs of localization error for the NLoS scenario obtained from the verification of the proposed performance-evaluation approach using the commercial localization system. (**a**) BW of 5 KHz; (**b**) BW of 25 KHz; (**c**) BW of 3125 KHz.

Figure 9 and Figure 10 depict the CDFs of localization error for the LoS and the NLoS scenarios, respectively.

Some observations can be obtained from Figure 9:

- It can be observed from Figure 9a,b that, with respect to all the four levels of SNRs, the median localization errors are less than 2.5 km for both BWs of 5 and 25 KHz. Moreover, 90% samples own the localization error of less than 4 km with respect to all the levels of SNRs for both BWs of 5 and 25 KHz.
- It can be observed from Figure 9b that the median localization error increases with the SNR decreasing.
- In Figure 9c, the median localization error for the BW of 3125 KHz is less than 0.05 km, which is much less than that for both BWs of 5 and 25 KHz. This reasonably occurs due to a larger BW providing a higher resolution for distinguishing two close wave paths, and therefore the localization system can utilize the multi-paths more accurately.
- Some cases of the CDFs do not exist, e.g., the SNRs of 8, 30 dB for the BW of 3125 KHz. Since the system may locate the source according to the larger delays of the DS paths caused by the ground scatterers rather than the LoS paths due to the time synchronization errors, a large localization error causes the system to stop outputting the source location estimates.

Some observations can be obtained from Figure 10:

- The median localization errors are respectively less than 1.5, 1.75 and 1 km for the BWs of 5, 25 and 3125 KHz with respect to all the specific levels of SNRs. Moreover, 90% samples own the localization errors of less than 2.25, 2.25 and 1.5 km for the BWs of 5, 25, and 3125 KHz, respectively, with respect to all the specific levels of SNRs.
- Some cases of the CDFs do not exist, e.g., the SNRs of 8, 30 dB for the BW of 3125 KHz. No output of the source location estimates can be a reminder of the system deployment, i.e., the TDoA sensors to the greatest extent are configured in an LoS environment instead of a complicated NLoS one.

### 6.2. Sensor Placement for a Realistic Area

TDoA localization performance is sometimes influenced by a topological structure of the sensors in an area which is a so-called “sensor placement” [48]. The wave propagation characteristics from the sources to the sensors are unique for one type of sensor placement in the area. For another type of sensor placement, the characteristics may vary since the waves interact with different objects causing the variations of propagation mechanisms, which eventually has an impact on the localization performance.

**Figure 11:**
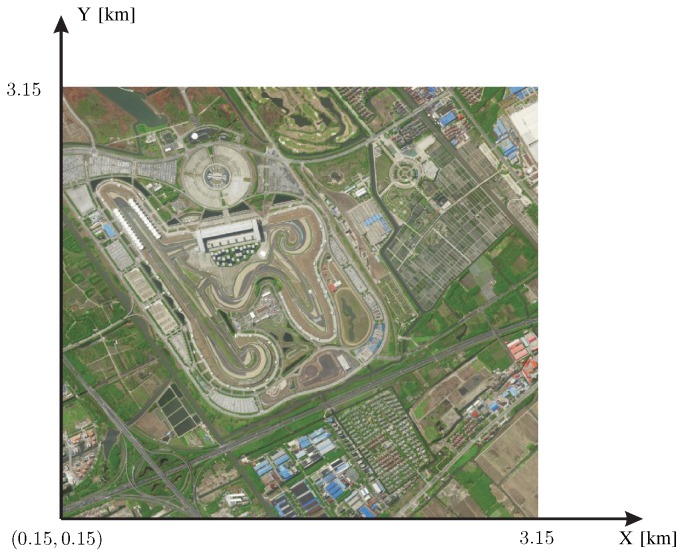
The satellite map of a realistic area of an automobile circuit for the verification of the proposed performance-evaluation approach in terms of the sensor placement.

Totally three types of sensor placement are configured for the verification here. The carrier frequency, the transmitted signal BW, the number of frequency points and the SNR are set as 1 GHz, 250 KHz, 100 and 20 dB, respectively. Figure 11 shows the satellite map of a realistic 3 km × 3 km area of an automobile circuit.

For the coordinate system of the satellite map, the location coordinates of the three sensors labelled with “Rx1”, “Rx2” and “Rx3” are shown in Table 2.

The 100 source candidates are uniformly placed in the area. The spacing between two adjacent sources is set as 300 m along both X and Y axes, and the Z component of the coordinates for all these sources is set as 1.5 m. In terms of the propagation graph simulation for the verification here, the scattering loss coefficients *S* for smooth and rough surfaces are set as 0.2 and 0.6, respectively. The parameter dS is set as 0.1 and 0.4 m${}^{2}$ for smooth and rough surfaces, respectively. 400 noise realizations are utilized to calculate one value of RMSLE.

**Figure 12:**
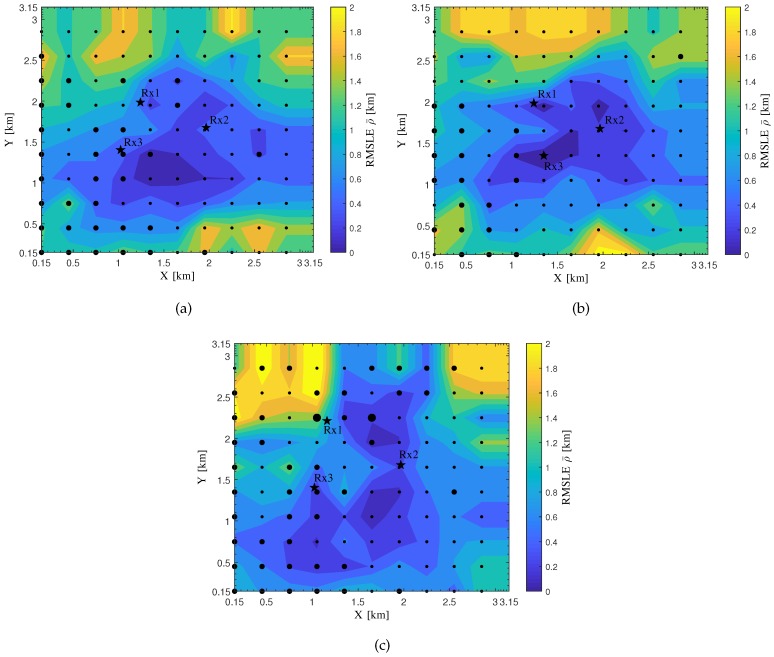
The RMSLE heat maps in terms of the three types of sensor placement for the realistic automobile circuit obtained from the verification of the proposed performance-evaluation approach using the commercial TDoA localization system. The sensors are marked with pentagrams while the sources with dots. (**a**) The first type of sensor placement; (**b**) The second type of sensor placement; (**c**) The third type of sensor placement.

Figure 12 shows the RMSLE heat maps for the realistic area of the automobile circuit obtained from the verification of the localization system under test, in which the heat maps vary with the different types of sensor placement.

In Figure 12, the dot size for a source is set the largest if an LoS path exists between the source and each sensor while the smallest if no LoS paths exist. In most cases here, zero or one LoS path exists between the source and all the sensors for the three types of sensor placement. Based on these results, the second type of sensor placement can be selected if one would like to obtain the lowest RMSLE in the area enclosed by the three sensors.

## 7. Conclusions

In this paper, we present a performance-evaluation approach for practical TDoA localization systems for an area with a complicated environment. The proposed approach has been achieved with three units. In terms of the software processing unit, the radio propagation graph is applied through a simulation to obtain the CIRs between a source to be located and the sensors of a TDoA localization system to be tested, for an area to be loaded as a digital map. The CIRs are combined with the transmitted signal of the source to emulate realistic signals received by the TDoA sensors in the baseband. With regard to the hardware transmitting unit, a multi-device peripheral suite is constructed with the accurate time synchronization among the RF devices, which simultaneously sends the multi-link emulated received signals on a radio carrier to the localization system under test. The maximum error of the time synchronization among the devices in our case is less than 1.5 ns through the evaluation using an oscilloscope. For the performance analyzing unit, the statistical evaluations are made based on the localization errors obtained calculating the distances between the estimates from the system and the ground truth of the source location. An RMSLE heat map elaborates the distribution of localization errors versus a group of source locations, which helps in placing the system sensors in the area to attain the optimum localization performance or assists in comparing two systems in terms of the whole area. The proposed approach is verified for both LoS and NLoS scenarios with the transmitted signal bandwidth and signal-to-noise ratio being variable, as well as for three schemes of sensor placement in an automobile circuit. Through the verified results, we have proven the usability of the proposed performance-evaluation approach.

## Figures and Tables

**Table 1:** The parameters used for the verification of the propagation graph simulation of the proposed performance-evaluation approach.

Parameter	Value	Unit
Carrier frequency	3600	MHz
Signal bandwidth	10	MHz
Number of frequency points	2001	-
dS for smooth surfaces	0.49	m${}^{2}$
dS for rough surfaces	0.81	m${}^{2}$
*S* for smooth surfaces	0.35	-
*S* for rough surfaces	0.55	-
Signal-to-noise ratio $r_{e}$	20	dB
Number of noise realizations $N_{s}$	120	-

**Table 2:** The sensor locations in terms of the three types of sensor placement in unit of meters.

	Type 1			Type 2			Type 3		
	Rx1	Rx2	Rx3	Rx1	Rx2	Rx3	Rx1	Rx2	Rx3
X	1241	1966	1022	1241	1966	1350	1160	1966	1022
Y	1985	1677	1403	1985	1677	1350	2212	1677	1403
Z	7.5	7.5	7.5	7.5	7.5	1.0	7.5	7.5	7.5
